# Pain Assessment in Goat Kids: Focus on Disbudding

**DOI:** 10.3390/ani13243814

**Published:** 2023-12-11

**Authors:** Kavitha Kongara, Preet Singh, Dinakaran Venkatachalam, John Paul Chambers

**Affiliations:** 1 School of Veterinary Science, Massey University, Tennent Drive, Palmerston North 4442, New Zealand; p.m.singh@massey.ac.nz (P.S.); j.p.chambers@massey.ac.nz (J.P.C.); 2 Ministry for Primary Industries, Wellington 6011, New Zealand; dinakaran.venkatachalam@mpi.govt.nz

**Keywords:** goat kids, disbudding pain assessment, physiology behaviour

## Abstract

**Simple Summary:**

Disbudding is a routine husbandry procedure performed in goat kids in their first few weeks of life. Behavioural and physiological changes following the procedure suggest significant pain and distress to the goat kid, which is a welfare concern. Pain assessment is fundamental to implementation of effective pain treatment and/or management protocols. This review provides details on pain assessment methods in goat kids following different methods of disbudding. Commonly used pain assessment methods in other young farm animals were also included.

**Abstract:**

Farm animals are routinely subjected to painful husbandry procedures for various purposes. Goat kids are disbudded to improve goat welfare and to ensure safety of other livestock, farm personnel, attending veterinarians and for various other production and managemental procedures. Disbudding is commonly performed on dairy goat farms, in kids under 3 weeks of age. Many scientific studies reported physiological and behavioural changes indicating pain and distress following disbudding, and this can be a significant cause of welfare compromise in goat kids. Recognition and measurement of pain is important to treat and/or manage pain and distress following painful procedures. This review focuses on pain assessment in goat kids following disbudding, using both physiological and behavioural measures. As only a limited information is available on the topic of interest, relevant studies in other young farm animals have also been discussed to compare the status quo in goat kids.

## 1. Introduction

Farm animals are routinely subjected to painful husbandry procedures for various purposes. Goat kids are disbudded to reduce injury to other goats in the flock, farm personnel, and attending veterinarians. Hornless goats are also less likely to get their heads entangled in equipment such as milking machines. Disbudding is commonly performed on dairy goat farms, in kids under 3 weeks of age. Many scientific studies reported physiological and behavioural changes indicating pain and distress following disbudding, and this can be a significant cause of welfare compromise in goat kids. Accurate recognition and assessment of pain is important to implement correct treatments at appropriate doses and intervals to ensure animal wellbeing [1]. The ability to report one’s own experience of pain and its intensity helps in developing effective management strategies in humans. Pain assessment in pre-verbal infants, people with cognitive impairment and animals is often challenging. In most instances, pain is assessed based on observation of animal’s behaviour [1]. Although this approach is useful in clinical situations, it can be difficult to assess subtle signs of pain in prey animals, such as sheep and goat, that do not manifest overt signs of pain [2].

Physiological and behavioural measures that are likely to indicate pain and/or nociception and related distress have been developed and used for assessment of pain and the efficacy of analgesic strategies in animal studies. The main aim of this review is to describe the methods of pain assessment in goat kids following husbandry procedures such as disbudding. Commonly used pain assessment methods in other young farm animals was also included, as little information is available in goat kids compared to calves, lambs, and piglets.

## 2. Physiological Measures

### 2.1. Plasma Cortisol, Glucose and Lactate

The physiological variable that has been historically studied to assess the pain related distress following routine husbandry procedures is the total plasma cortisol [3]. Cortisol is a hormone, which is released in high concentrations into circulation after activation of hypothalamo-pituitary- adrenal (HPA) axis in response to stressful conditions including pain [4]. It thus gives an indication of distress, rather than pain directly. Increases in plasma cortisol concentrations following disbudding and other painful husbandry procedures such as castration and tail docking, and its alleviation by analgesic administration has commonly been used to assess pain in calves, lambs, and goat kids [5,6,7]. The degree of plasma cortisol response was also used to compare the pain intensity between different methods of goat kid disbudding [8]. Disbudding of kids with liquid nitrogen or caustic paste has been found to cause more severe changes in cortisol, indicating intense acute distress, and possibly pain, compared to cautery disbudding [8].

To measure plasma cortisol, blood samples are commonly collected from the jugular veins and cortisol concentrations are measured using different assay techniques, including the enzyme-linked immunosorbent assay and radioimmunoassay [5]. Although many studies have used plasma cortisol for the assessment of pain induced distress, there are some caveats to this method. Plasma cortisol levels were also influenced by diurnal rhythms [9] and stressors that are not associated with pain such as animal handling [10], feeding [11], and sexual excitement [12]. Trends in plasma cortisol concentrations measured from blood samples collected before and at multiple time points after a specific procedure would give a more reliable estimate of the stress associated with pain following a noxious procedure in production animals (although blood sampling will increase plasma cortisol). In analgesia studies, comparing the cortisol levels between sham-handled and treatment groups, and correlating cortisol response with other pain assessment measures such as pain behaviours, would make plasma cortisol evaluations a more valid way to estimate the efficacy of analgesic drugs [13]. It should be noted that some drugs which are used as analgesics in ruminants, such as alpha 2 adrenergic agonists, probably have a direct depressant effect on the adrenal glands in goats, as they do in pigs [14].

Plasma metabolites, such as glucose and lactate, were measured in conjunction with plasma cortisol following disbudding of goat kids [8,15]. These blood constituents were measured based on the hypothesis that increased secretion of cortisol in response to pain induced distress stimulates mobilization of glycogen, which results in increased production of glucose and lactate [16]. None of the studies found significant changes in plasma lactate and glucose despite a significant elevation of plasma cortisol after disbudding. The researchers opined that these two metabolites are not as useful as plasma cortisol responses to acute pain associated with disbudding.

### 2.2. Autonomic Responses

Other physiological measures resulting from the activation of sympathetic nervous outflow in response to noxious stimulation, such as heart rate, respiratory rate, blood pressure and body temperature can be monitored to assess pain following disbudding in goat kids [6]. Although changes in these variables are influenced by the type of noxious stimulus based on the method of husbandry procedure; in general, animals in pain often have an increase in these variables [17,18]. A major limitation to the use of the sympathetically driven variables, similar to cortisol, is that changes are not necessarily specific to pain [19], and are usually limited to acute pain responses and hence less useful for the assessment of longer lasting pain. Also, changes in these autonomic variables have poor correlation with pain perception as these are modulated by centres of the brain stem below the level of the higher centres involved in cognitive pain perception [1].

### 2.3. Electroencephalography (EEG)

The EEG is a record of the spontaneous electrical activity of the cerebral cortex. It is an objective tool for assessment of neural (sensory) processing of noxious stimuli, which is known as nociception. EEG provides a direct and quick profile of cortical neuronal activity in response to noxious stimulation in animals. The characteristic configuration of neurones (particularly pyramidal type) in the layers of the cerebral cortex leads to formation of electrical vectors that facilitates transmission of electrical currents. The EEG is the far-field potential (electrical field created by inherent neuronal activity and recorded away from them) of the vector currents recorded using electrodes on the surface of the head [20]. Fast Fourier transformation, a mathematical procedure, converts the raw EEG signal into its component sine waves of different frequency, characterized by corresponding amplitude. Thus, the power spectrum generated is simply a distribution and derivation of spectral EEG variables from the corresponding frequencies and amplitudes [20]. There are also a number of other proprietary methods of analysing raw EEG data, which are used in instruments designed for people.

Changes in the EEG power spectrum have been used for the quantification of nociception in minimally anaesthetised red-deer, lambs, and calves following noxious procedures such as velvet antler removal, castration, and slaughter through a ventral neck-cut, respectively [21,22,23]. Changes in frequency spectra of EEG in lightly anaesthetized animals (with halothane, which is minimally analgesic) reflect changes in activity of cerebral cortex in response to pain perception [22,23]. This concept is further supported by studies in goats that did not find a difference in EEG spectral frequencies between conscious animals and those that were lightly anaesthetised during slaughter [24]. Advantages of the minimal anaesthesia model developed for recording EEG for quantification of pain in animals include the minimization of the impact on the EEG of extraneous electrical activity (such as from muscles) and loss of conscious pain perception by study animals, thereby reducing suffering from pain during noxious stimulation [20].

In goats, EEG power spectra have been used to delineate loss of awareness from cognitive perception of pain following the application of various techniques for euthanasia and pre-slaughter stunning [25]. Also, there have been studies that described the EEG frequency band analysis to find the effect of pre-slaughter stress, experimental pain and ontological changes in brain activity in goats [26,27,28]. So far, no studies are available on the use of EEG spectral frequency changes to quantify pain associated with husbandry procedures such as disbudding/dehorning in goats.

Although an electroencephalogram can non-invasively capture the sensory neuronal processing of noxious stimuli and indirectly reflect cortical pain perception, it may not represent the motivational states such as aversiveness, fear and anxiety associated with pain perception, all of which are subjective experiences. Another limitation to the use of the EEG is that it can only be used for acute pain assessment, as an animal can be kept minimally anaesthetized for only a limited amount of time. A combined approach such as concurrent evaluation of cognitive pain perception manifested by behavioural changes will be more reliable for pain assessment using EEG in farm animals.

### 2.4. Mechanical Nociceptive Threshold Testing

Mechanical stimuli, such as pressure, are usually applied to the skin, and can be used to produce quantifiable nociception or pain in farm animals, including goats. The degree of mechanical pressure that an animal can tolerate before showing an avoidance (behavioural) response or withdrawal reflex is defined as a mechanical nociceptive (pressure) threshold of that animal [29,30]. Mechanical stimuli are used to induce experimental pain to test or compare analgesics in normal animals. In animals subjected to noxious procedures, these stimuli are used to test the pain sensitivity at the site of tissue damage (wound site) and/or areas surrounding the wound. A variety of commercial, hand-held algometers are available (e.g., Force one FDIX 50, Wagner Instruments, Riverside, CT, USA; ProdPlus algometer, TopCat Metrology Ltd., Ely, UK), which are validated for use in various farm animal species, including goats [18,31,32]. Mechanical stimulation of the wound area (injured tissue at the disbudded area) can also be performed to test the tactile sensitivity, using von Frey filaments [31,33,34]. Wounds remained sensitive to tactile stimuli for at least 75 h after disbudding in calves [33].

Mechanical nociceptive thresholds are usually measured in Newtons (N) or Kilogram Force (Kgf). Force is applied perpendicular to the skin surface at a constant rate and in gradual increments to avoid a sudden, jerky increase in readings without corresponding attainment of threshold manifested by signs of discomfort specific to the species being tested. Head withdrawal with a specific ear flick has been noted as the end response by goat kids being tested at the disbudded site [30,34]. To attain a good correlation between threshold values and the amount of pressure applied, selecting an algometer probe that matches the size of the animal is also important. A round rubber tip probe of 1 cm^2^ has been used on a Force One Wagner algometer [30], and a 2 mm diameter metal probe has been used on a ProdPlus algometer in disbudded goat kids [32,34]. Also, other factors such as age and body weight of the animal should be considered while testing nociceptive thresholds for a study in piglets; age and body weight were demonstrated to affect the pressure threshold testing responses [35]. In goat kids, pressure algometry has been used to compare the pain sensitivity between three different methods of disbudding, and cautery disbudding was found to cause less acute pain hypersensitivity than the caustic paste and cryosurgery methods [30]. In other studies, it was used to find the welfare benefits of alternative methods of disbudding [34] and the duration of wound hypersensitivity after hot-iron disbudding in goat kids [32]. In the study by Frahm et al. [34] development of acute mechanical hypersensitivity around the horn buds following the injection of clove oil or isoeugenol has been reported [34]. Tissue irritation by the injected substances was suggested as the potential cause of heightened pain sensitivity and, therefore, the welfare benefits of the method over hot-iron disbudding are questionable. Persistent mechanical hypersensitivity throughout the wound healing period (average 7-weeks) has been reported by Alvarez et al. [32].

Animal handling prior to the application of the stimulus has the potential to influence the threshold readings. A good human–animal relationship, prior habituation of the animal to the device, and testing in home pens close to dam/other pen mates can minimize the confounding effect of handling stress on threshold measurements [34].

Other types of nociceptive stimuli that have been used to test the effect of local anaesthetic (LA) administration prior to goat kid disbudding were pin pricks [36]. The observer gently pricked the skin around the horn buds with a needle point at approximately 30 s to 1 min interval until no response is observed to confirm analgesia after infiltration with LA. This qualitative analgesia testing method is rapid and practical to use in field conditions, and reliable to assess the onset of analgesia prior to disbudding.

In summary, all the devices and methods developed to test the mechanical nociceptive thresholds of farm animals let us assess the level of pain hypersensitivity, efficacy of analgesics and/or analgesic approaches, and development of ‘plasticity’ in the nervous system, following noxious husbandry procedures in field conditions.

### 2.5. Infra-Red Thermography

Infra-red thermography (IRT) is a non-invasive method of recording infrared radiation emitted by bodies, i.e., heat [37]. It can measure changes in surface temperature due to activation of autonomic nervous system in response to stress. A stress-induced secretion of catecholamines causes an increase in internal body temperature called stress induced hyperthermia, and also causes a reduction in blood flow in the skin around eyes. A drop in eye temperature, assessed using IRT, has been reported to be associated with the onset of acute pain in calves following hot-iron dehorning [38,39]. Sympathetically mediated vasoconstriction in response to acute pain has been proposed to be the cause of the drop in eye temperature. The IRT recording can be taken at a distance from the animal without a need to contact/restrain the animal. This overcomes the effect of animal handling stress on actual data, which is the drawback associated with the measurement of other physiological (and autonomic) variables in response to stress and pain [38] Care must be taken that the hair coat must be free of dirt, grease and foreign material while obtaining IRT images of the area of interest [39].

A study in disbudded goat kids used IRT to take the images of the horn bud (~3 cm diameter around horn bud), and surface skin temperature was calculated from a ~2 cm diameter area around the horn buds [8]. Disbudded goat kids were found to have a higher skin surface temperature around the horn buds than sham kids. Another study has used IRT to assess the wound inflammation and surface temperature during healing after cautery disbudding of goat kids [32], and reported that the necrotic tissue formed during the inflammatory phase was hotter than epithelium. Studies are required to find the use of the IRT to evaluate the effect of analgesic agents and strategies after disbudding in goat kids.

### 2.6. Biomarkers

Immunological, inflammatory and pain biomarkers were measured in tissues and body fluids of young farm animals following husbandry procedures [31,40,41,42]. Acute phase inflammatory proteins such as haptoglobins were measured in goat kids disbudded using different methods [8]. Clove oil injection under the horn bud caused a significant increase in serum haptoglobin concentrations 24 h after treatment, which indicates marked inflammation associated with the method of disbudding [8]. Measurement of immunoreactive proteins such as β-endorphin concentration in the blood plasma has been shown to be a useful method of acute pain assessment following hot-iron disbudding of goat kids [43]. With the advent of new molecular biological techniques in animals, it has been possible to demonstrate the efficacy of non-steroidal anti-inflammatory drugs in suppressing the expression of mRNA (in peripheral leucocytes of a blood sample) for inflammatory cytokine and nociceptive marker genes in calves disbudded by thermocautery [42]. More advanced techniques such as plasma proteome analysis allowed identification of candidate protein biomarkers that are associated with nociceptive and inflammatory processes following surgical dehorning of calves [44]. Biomarkers such as these may be useful in the future to assess various methods of pain mitigation for husbandry procedures in farm animals including goats.

## 3. Behavioural Measures

Changes in normal behaviour are commonly observed in animals in response to painful stimuli. Hence, monitoring normal behavioural patterns and deviations from these is a significant component of pain assessment in animals [45]. Species-specific behavioural changes that can be identified and quantified have been used to evaluate pain and analgesia in goat kids, lambs, piglets, and calves following routine husbandry procedures [4,6,46]. A person assessing pain behaviours, either directly from an animal or indirectly from video recordings, should be familiar with the animal’s normal behavioural patterns, have adequate training and experience in recognition of changes in normal behaviours in response to noxious procedures [47,48].

A multitude of factors including severity of insult to the tissues and neuronal pathways influence the manifestation of pain behaviours by animals. Age, previous pain experience, social hierarchy in the herd, human presence, environmental conditions are among the others [49]. In young farm animals such as goat kids, lambs and calves, a significant alteration in their normal behaviour, body posture, locomotor activity, orientation toward dam, and response to manipulations have been used as a basic template for the behavioural assessment of pain and analgesia following husbandry procedures, including disbudding.

There appears to be two phases to goat kids’ behaviour in response to hot iron disbudding—acute avoidance type behaviours in responses to application of the iron and abnormal behaviour in the subsequent hours. Behavioural indicators of acute stress and pain during cautery disbudding in goat kids have been first reported by Alvarez et al. [6] Struggles (kicks), such as a vigorous movement of the legs and attempts to escape, and vocalisations in the form of bleats with an open or closed mouth were reported. A change in the frequency of behaviours along with plasma cortisol levels was used to find the effect of analgesic treatment on disbudding pain [7].

In a sham controlled trial, Hempstead et al. [50] recorded behavioural changes associated with cautery disbudding of female Saanen dairy goat kids. Video recordings of pre- and post-treatment behaviours were analysed by a trained and an experienced observer to build an ethogram of behaviour patterns. Although the frequency of 11 behaviours in the ethogram was chosen to study, individual behaviours such as head shaking, rubbing, scratching, and body shaking were significantly different between treated and control kids. Due to a large inter-individual variation, self-grooming behaviour did not reach statistical significance despite an apparent increase in its frequency in the disbudded group. Individual variation in a behavioural response, within the same species, to a noxious procedure is one important confounding factor in pain assessment studies. This could be due to differences in pain perception and expression between individual animals [51]. The following table (Table 1) provides an overview of behavioural changes in goat kids following different methods of disbudding.

In addition to the assessment of frequency of specific behaviours, some studies used a visual analogue scale (from 1–10) to score pain behaviours to evaluate the efficacy of anaesthetic and analgesic treatments administered to goat kids [56,57]. A summary of the pain behaviours was used to assign a score from the scale. Also, a vocalisation score, which is the total number of vocalizations during the disbudding procedure for both horn buds, was used to compare the alternative methods to cautery disbudding [58].

From these studies in goat kids, it is evident that disbudding induces specific changes in behaviour, which include increased frequency of struggles, vocalisations, head shaking, scratching, and rubbing, body and tail shaking, lying bouts and duration. Changes in these behaviours have been used to evaluate relative noxiousness of different (alternative) methods of disbudding and efficacy of anaesthetic and analgesic protocols in various studies.

### Facial Grimace Scale

Due to the limitations of behavioural methods (such as laborious and time-consuming video and audio recording and analyses and the need for specialised equipment), and physiological measures (such as expensive laboratory analytical procedures), Lou et al. (2020) developed a goat kid grimace scale for pain assessment following thermal disbudding [59]. It is a pain assessment method that enables instantaneous identification and assessment of changes in facial expression after a noxious procedure [60]. The basic template for the scale has been drawn from sheep and lamb grimace scales [61,62] and adapted for a goat face. Orbital and lip tightening, nostril dilatation and change in ear position have been scored on a 3-point scale (0 = Not Present, 1 = Moderately Present, and 2 = Obviously Present), from photographic images, to find the effect of analgesic administration and thermal disbudding in goat kids. Orbital tightening and ear position scores were found to be more reliable indicators of treatment effects than lip tightening and nostril dilation. More research needs to be conducted to further explore the validity of this facial grimace scale in goat kids.

## 4. Conclusions

Both the behavioural and physiological systems are involved in the response to stress and pain, and in majority of research studies, a combination of both methods is used in pain assessment. Individual behaviours or an ethogram in conjunction with plasma cortisol measurement has been the basic pain assessment paradigm in a large number of goat disbudding studies. Some studies used other physiological methods such as mechanical nociceptive threshold testing, infrared thermography and plasma inflammatory marker analyses, heart rate and body temperature changes, and body weight gains, etc. Only one study used a facial grimace scale in combination with plasma cortisol evaluations in disbudded goat kids. Using a combination of pain assessment methods allows to offset some of the disadvantages associated with each technique and to gain as much accurate and comprehensive information as possible from an experimental study [63]. In clinical practice, a combination of pin prick testing for analgesia before disbudding and behavioural assessment afterwards is probably most practical.

## Figures and Tables

**Table 1:** Individual behaviours and ethograms described in various studies investigating pain and pain mitigation strategies in disbudded goat kids.

Title of the Study	Behavioural Indicators Used	Other Variables Measured
Physiological and behavioural alterations in disbudded goat kids with and without local anaesthesia [6].	Struggles and vocalisations	Plasma cortisol, Heart rate and respiratory rate pre- and post (up to 4 h) disbudding
Evaluation of alternatives to cautery disbudding of dairy goat kids using behavioural measures of post-treatment pain [52].	Head and body shaking, head scratching, feeding and self-grooming noted from video recordings 24 h pre- and post-disbudding	Accelerometery measures such as lying bouts and duration recorded 24 h pre- and post-disbudding
Effect of isoflurane alone or in combination with meloxicam on the behavior and physiology of goat kids following cautery disbudding [53].	------Do----	Plasma cortisol, glucose and lactate measured pre- and post (up to 120 min) disbudding
Acute cortisol and behavior of dairy goat kids administered local anesthesia, topical anesthesia or systemic analgesia prior to cautery disbudding [54].	Rump movements, tail shakes and vocalisations recorded during disbudding	Plasma cortisol levels measured pre- and post-disbudding
Can Isoflurane and Meloxicam Mitigate Pain Associated with Cautery Disbudding of 3-Week-Old Goat Kids? [15]	Head and body shaking, head scratching, feeding and self-grooming noted from video recordings 1 h pre- and post-disbudding	Plasma cortisol, glucose and lactate measured pre- and post (up to 120 min) disbudding
Evaluation of Pain Mitigation Strategies in Goat Kids after Cautery Disbudding [55]	State events (head scratching, self-grooming, allogrooming, feeding/drinking, exploration, standing, lying, social play, etc.) and point events (vocalisation, shaking, tail movements, stretching, etc.) in an ethogram built using BORIS * software (https://www.boris.unito.it/ accessed on 1 May 2023) from a 3 h video recording after disbudding.	--------------

* BORIS; Behavioural Observation Research Interactive Software.

## Data Availability

Data is contained within the article.
